# A macroscopic Washburn approach of liquid imbibition in wood derived from X-ray tomography observations

**DOI:** 10.1038/s41598-022-05508-0

**Published:** 2022-02-02

**Authors:** Patrick Perré, Dang Mao Nguyen, Giana Almeida

**Affiliations:** 1 CentraleSupélec, Laboratoire de Génie des Procédés et Matériaux, SFR Condorcet FR CNRS 3417, Centre Européen de Biotechnologie et de Bioéconomie (CEBB), Université Paris-Saclay, 3 Rue des Rouges Terres, 51110 Pomacle, France; 2 INRAE, AgroParisTech, UMR SayFood, Université Paris-Saclay, 91300 Massy, France

**Keywords:** Permeation and transport, Plant physiology, Wetting, Structure of solids and liquids, Biological physics

## Abstract

Imbibition of water and silicone oil in poplar and spruce is investigated at the anatomical level by X-ray tomography observations and at the macroscopic level by imbibition kinetics. Imbibition mechanisms depend on both liquid and species. In poplar, oil penetrates vessels with a small contact angle, consistent with the value measured on solid wood (ca. $20^{\circ }$). Surprisingly, no direct penetration of water was observed in vessels. The large contact angle for water blocks the capillary rise at the scars between vessel cells. In spruce, oil and water penetrate primarily in latewood, where bordered pits remain open. Subsequently, water slowly invades the rest of the growth ring, while transversal migration is quasi-absent for oil. These 3D observations were quantified to feed a simple imbibition model that satisfactorily simulates macroscopic imbibition kinetics. A 1D approach is sufficient for oil imbibition while a 2D approach is required for water, revealing dual scale effects.

## Introduction

The vascular system of trees plays crucial roles during the plant’s life, namely cohesion-tension for sap ascent, resistance to drought and recovery after cavitation^1–5^. This vascular system also acts for liquid movement in wood as a material, through the important role it has in drying, processing, durability, weathering and building pathology^6–10^. Even though the phenomena that occur in the vascular system in trees and wood products are not identical, the biophysics and bio-based materials fields can benefit from the study of liquid imbibition in wood.

Over several decades, many studies were devoted to wood-water relationships and the mechanism of water transfer in wood^11–15^. The need to dry wood before use has been a strong driver in the research on water transfer in wood^16,17^. The formulation and computational simulation of transfers in wood benefited from the general progress in studies of coupled and simultaneous heat, mass and momentum transfer in porous media that started at the beginning of the last century^18^. Even though the physics embedded in this formulation derives from the standard conservation laws^19,20^, the challenge is to overcome the problems associated with structural dependencies and the complex geometries evidenced in the internal pore network within the medium. Typically, transport phenomena are formulated at the macroscopic level. The explanations assume that the variables are defined at the level of many pores and that the porous material can be represented as a fictitious continuum^21^. In this framework, it is possible to rigorously derive the macroscopic equations from microscopic balance equations using volume averaging^22–24^.

Nowadays, the formulation of coupled heat and mass in wood and its computational solution are well-established at the macroscopic level. These models are widely used, either to simulate 1D to 3D configurations, including with the effect of internal pressure or as a physical engine to account for the strong heat and mass transfer in materials characterization by an inverse method^25–31^. In terms of materials characterization, most norms and laboratory methods rely on measurements made on macroscopic samples^32–34^. This practice is consistent with a macroscopic formulation, and these measurements are likely to be used in computational tools to predict coupled heat and mass transfer in buildings.

However, more subtle phenomena taking place at the microscopic or molecular scale have important effects at the macroscopic level. These phenomena include non-Fickian behaviors, molecular relaxation, fading, and the permanent effects of the history of hydric loading and failure of local equilibria^35–37^. Unlike drying, imbibition of liquid does not involve any phase change, which drastically reduces the heat and mass coupling. Surprisingly, this simplification is also likely to give rise to complex dual-scale effects, in which the microscopic field depends on the history of the macroscopic field. In the absence of resistance to the heat transfer required for phase change, liquid migration becomes the sole resistance to transfer. Imbibition can, therefore, be very rapid in connected pores and then much slower in the remaining tissues, which leads to a dual-scale effect inducing the failure of the classical macroscopic approach^38–40^.

In order to be predictive, it is important to embed these dual-scale effects in the macroscopic formulation when manufacturing and using bio-based materials throughout their life. Fortunately, recent progress in experimental investigation provides outstanding tools able to investigate the relationships between microscopic and macroscopic scales^41^. Among them, nuclear magnetic resonance (NMR) is a bulk method able to distinguish and quantify different kinds of water present in wood, bound water and size of pores filled by free water^42–46^. Magnetic resonance imaging (MRI) combines NMR with field gradients and allows 3D imaging. The distribution of water in poplar was studied through 2D MRI^46^; the images showed the penetration of water along the growth rings with different rates due to the heterogeneous structure of this species. This technique was also applied to observe the water drainage of Douglas fir, where it proved that latewood was rapidly drained compared to earlywood because bordered pits are less prone to aspiration in latewood^47^. Neutron radiography is another method that allowed spatial fields to be obtained. Imbibition of free water was assessed using this method to follow liquid invasion in latewood and earlywood^48^. The images from neutron radiography were used to explain the mechanism of water transfers and indicate the dependent correlation in the progression of bound water and free water in the hardwood during water imbibition^49^. Note, however, that these observations do not necessarily prove a causal relationship.

Computed tomography is 3-D imaging method based on X-ray attenuation that was first used for medical imaging. Its improved spatial resolution, up to the sub-micrometric resolutions available in laboratory X-ray tomography, promoted this technology to material sciences^50–53^. Aided by relevant image processing, these techniques are extremely useful tools to describe the dynamics and mechanisms of liquid transport in porous materials by 4D imaging (3D spatial fields as a function of time). They also allow the liquid content in the materials to be quantified at a macroscopic scale under certain conditions^54–56^. Tomography was used to study the transport of water (imbibition or drying) in wood to determine the distribution of liquid water^57^. The variation of mass, mechanism, and dynamics in different directions (longitudinal, radial, and tangential) have been documented. Particularly, X-ray computer tomography was used to differentiate liquid water locations in wood during imbibition^40^. The water uptake in sapwood was faster than heartwood due to less aspiration of bordered pits in sapwood. For the same reason, water uptake occurs preferentially only in latewood in the case of heartwood^48^. Synchrotron facilities, which are capable of combining very high resolution with short acquisition times, have been successfully applied to the study of lignocellulosic products^58–60^.

Even though anomalous behaviors of the roughening of wetting fronts in spontaneous imbibition have been reported^61–63^, the Washburn-like scaling (imbibition height as a function of the square-root of time) is generally observed in porous media. Note, however, that deviations from this scaling law were observed in the case of carbon nanotubes^64^ and in the case of wood^40,65^.

The objective of this paper is to propose a suitable macroscopic modeling approach for liquid imbibition. It relies on the observation, by X-ray tomography, of the dynamics of imbibition for two fluids (water and silicone oil) and two species (spruce and poplar) to better assess the migration mechanisms in wood. An innovative method is integrated into a laboratory X-ray tomography to perform *in-situ* water and oil imbibition in wood. Besides, an experimental protocol was applied to small boards to follow the amount of liquid penetration in wood from the dry state. The observations gained at the anatomical level from these four case studies allow us to propose an imbibition model based on Washburn’s approach. The model predictions were successfully compared to macroscopic measurements of liquid imbibition. In the case of oil, a suitable 1D model is perfectly able to reproduce the macroscopic observations. The phenomena are more complex with water as imbibition is much slower than it should be. A recent paper suggested that the dynamic of bound water is the reason for the slowing down of fluid imbibition^66^. However, the characteristic time of the observed mechanisms is likely to let enough time for the bound water content to increase at the cell wall once liquid is present. In the present work, we simply explain the surprising shape of the meniscus in vessels by geometrical anatomical features that blocks the meniscii in vessels. This observation requires a 2D modelling approach that mixes the macroscopic longitudinal scale and a microscopic dimension. To the best of our knowledge, this is the first time the Washburn approach has been adapted to the anatomical features of wood to propose a model (1-D or 2-D, depending on the situation) that can predict the macroscopic experiments.

## Materials and methods

### Materials

Two species of contrasted properties were selected from the wood collection stored and cataloged in our research group. The poplar tree (*Populus euroamericana *Koster) comes from a forest in Auménancourt-le-Petit (France). The poplar was kindly provided by the Huberlant sawmill (Cormicy, France) who felled and cut the tree. The spruce tree comes from a plantation in the Le Châtaignier forest in Riotord (France). Both trees were obtained from a sawmill who had the permission to collect them. The bottom log of these trees were through and through cut. The boards were then dried and stored in a dry, temperature-controlled room. Experimental research and collection of plant material are in accordance with relevant institutional, national and international guidelines and legislation.

All samples were obtained from a single defect-free board with a straight grain angle and carefully cut along the longitudinal direction, in which the natural variability of wood is the lowest. The sample size and preparation method were adapted to the constraints of each imaging technique.

**Figure 1:**
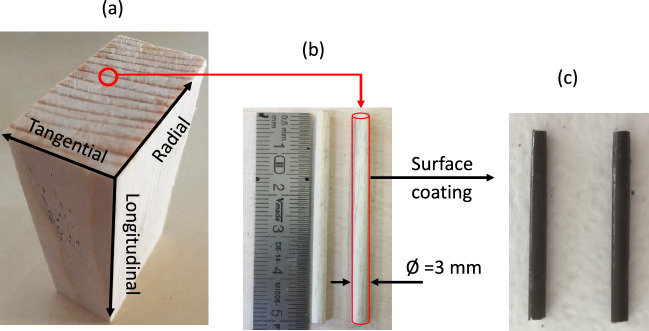
Sample preparation (**a**) macroscopic sample (Spruce here) prepared along the orthotropic directions: radial (R), 40 mm; tangential (T), 20 mm; and longitudinal (L), 100 mm, for macroscopic imbibition test; (**b**) small wood cylinder of 3 mm diameter and 50 mm length prepared for microscopic tests observed with the X-ray tomography, and (**c**) wood specimens sealed by a water-impermeable paint (aqua-stop) to avoid any capillary effect at the external face (photographs by M. Nguyen).

The experimental wood samples are collected from the boards along the orthotropic material directions: longitudinal (L = 100 mm), radial (R = 40 mm) and tangential (T = 20 mm) (Fig. 1a). These samples are dedicated to dynamic macroscopic imbibition tests using an electronic balance. Small cylinders with a diameter of approx. 3 mm and a length of approx. 50 mm (zoom of Fig. 1b), including both latewood and earlywood from the heartwood, are prepared for microscopic imbibition tests. The lateral surfaces of all specimens, both microscopic and macroscopic, are sealed parallel to the longitudinal direction with a water-impermeable paint (Aqua-stop) (Fig. 1c) to eliminate the capillary effects between the external wood surface and the water bath. The faces of macroscopic and microscopic samples in contact with the fluid were prepared using a sliding microtome to ensure that the anatomical elements are not closed by powder or fibers (Fig. S1).

The imbibition is performed with deionized water and a non-polar silicone oil with density, viscosity and surface tension equal to 997 kg m$^{-3}$, 0.001 Pa s, and 0.073 N m$^{-1}$ and 1000 kg m$^{-3}$, 0.02 Pa s, and 0.021 N m$^{-1}$, respectively, at $25\,^{\circ }\hbox {C}$. The oil was purchased from Sigma-Aldrich.

### Microscopic observation

**Figure 2:**
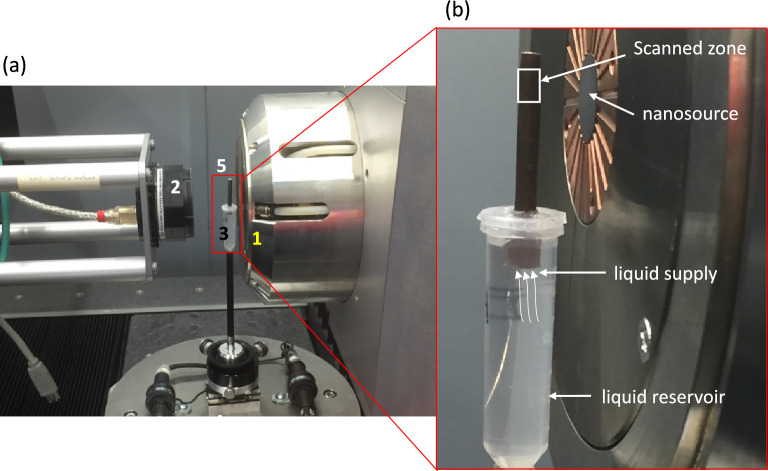
Laboratory-scale tomography used to study the mechanism of liquid transport in wood: (**a**) experimental setup, 1-X-ray nano-source with a tungsten filament, 2-CCD detector, 3-liquid provider, 4-rotation axis, and 5-wood specimen; and (**b**) scan area of the wood specimen in the case of silicone oil imbibition facing the nano-source (photographs by M. Nguyen).

Microscopic dynamics of liquid imbibition tests were performed using X-ray tomography (model EasyTom XL 150/160, RX-solutions, France). The tomography configuration was set up with a nano-source (tungsten filament), a CCD detector of 2004 $\times$ 1336 pixels, a voxel size of 3.6 $\upmu$m, and an exposure time of 1 s per frame (26 min to obtain 1100 projections for 3D reconstruction). The liquid container is directly integrated into the tomography system through a carbon tube that provides the water/oil during the experiment (Fig. 2). All tested samples were immersed in the liquid about 5 mm below the liquid level. The scanned area starts at ca. 20 mm and 5 mm above the liquid surface for silicone oil and water, respectively. The specimens were scanned every 30 min for silicone oil and every 2 h for water.

In order to perform the segmentation of the 3D representation of the sample obtained as a stack of 2D images by the reconstruction software, the data was processed by the Avizo software (Thermo Fisher Scientific, USA). In computer tomography, keeping in mind that the reconstruction generates noise, an accurate quantification is not an easy task when the contrast between phases is weak. The automatic thresholding methods proposed by Avizo failed, even for oil in poplar, the simplest configuration due to the large and uniform vessel areas and the good contrast between the cell walls and the oil. We therefore use a manual thresholding. After extracting subvolumes from the original datasets, the Segmentation Editor was applied to generate discrete label fields according to intensity ranges selected by the Thresholding tool. The CT number of oil is larger than that of cell walls and the very last high values of gray levels allow oil to be selected. This allowed us to generate nice images for this configuration. On the contrary, the contrast between cell walls and water is very low, water ranging between the image background and cell walls. In this case, it is impossible to select a threshold value for water that is able to avoid either the inclusion of cell walls or the omission of some water. As the manual thresholding is operator dependent, no quantification of liquid content will be presented in the microscopic tests.

### Macroscopic tests

The macroscopic dynamics of liquid imbibition were assessed in the longitudinal direction using a microbalance with the macroscopic samples as shown in Fig. 1a. All specimens were pre-conditioned at 44% relative humidity (RH) and $22\,^{\circ }\hbox {C}$ until equilibrium before the test. The obtained equilibrium moisture content (EMC) equals 12.2%($\pm 0.2\%$) and 11.4%($\pm 0.1\%$) for poplar and spruce, respectively. The open bottom surface of the sample was placed about 5 mm below the liquid level. The container was covered by aluminum foil to avoid evaporation in the case of water. The sample is hung on the microbalance via a small wire. The mass evolution of liquid entering the sample is continuously recorded by a microbalance through an automatic recording system. The mass collected by the balance must be corrected for buoyancy to obtain relevant results; buoyancy evolves during the test as the liquid penetrating the sample lowers the liquid level. The corrected mass uptake, $\Delta m$, is further converted to the equivalent height of rise $h_{eq}$:

1 $$\begin{aligned} h_{eq} = \frac{\Delta m}{\rho S} \end{aligned}$$

where $\rho$ is the fluid density, and *S* is the sample cross section.

### Contact angle

Contact angle measurements were made in the longitudinal-radial plane for both species. Wood surfaces were prepared using a sliding microtome (HM 450, Thermo Scientific) and maintained at ambient conditions. The contact angle was measured by a Teclis Tracker tensiometer (Teclis Instruments, France). Water and silicone oil drops of $3\upmu$L were controlled by the tester’s syringe system during the tests. All contact angles were determined under ambient laboratory conditions at ca. $19\,^{\circ }\hbox {C}$. Due to wood anisotropy, the drops spread differently along the longitudinal (L) and radial (R) directions. Thus, images were grabbed in the “L” and “R” directions. Tests were performed in duplicate, and two angles (right and left) were determined for each image.

### Modeling

The well-known Lucas-Washburn approach^67,68^ was primarily derived to model capillary flow in a tube but can easily be extended to imbibition in a porous medium. For a vertical tube in contact with the liquid at time $t=0$, this equation of the original paper considers three forces: capillary pressure, gravity and viscosity. Fluid inertia, which acts at very short times, is neglected.

2 $$\begin{aligned} dV = \pi R^{2} dh = \left( \frac{2 \sigma cos(\theta )}{R} -\rho g h\right) \frac{\pi R^{4}}{8 \mu h}dt \end{aligned}$$

The gas viscosity is assumed to be negligible hereafter. After integration, one obtains a relation between time *t* and meniscus height *h*^68^:

3 $$\begin{aligned} t=-\frac{8\mu }{\rho g R^2} \left( h + h_{max} \ln \left( 1-\frac{h}{h_{max}}\right) \right) \end{aligned}$$

where $$h_{max} = 2 \sigma cos(\theta ) /(\rho g R)$$.

However, as wood tissues are not simple bundles of capillary tubes, Eq. (2) much be modified to involve the key characteristics of a porous medium: porosity, $\varepsilon _g$, intrinsic permeability, *K* and equivalent pore radius, $R_{eq}$:

4 $$\begin{aligned} dV = \varepsilon _g dh = \left( \frac{2 \sigma cos(\theta )}{R_{eq}} -\rho g h\right) \frac{K}{\mu h}dt \end{aligned}$$

which gives, after integration:

5 $$\begin{aligned} t=-\frac{\mu \varepsilon _g }{\rho g K} \left( h + h_{max} \ln \left( 1-\frac{h}{h_{max}}\right) \right) \end{aligned}$$

In Eq. (5), the height *h* is the actual height in the pore, and the right value corrects the capillary pressure for the effect of gravity. The equivalent height, $h_{eq}$, should be used to compare with the height determined by the macroscopic experiments. This value, which represents the total volume of soaked liquid spread over the whole section of the sample, is simply obtained by multiplying the height by the active porosity:

6 $$\begin{aligned} h_{eq} = \varepsilon _g h \end{aligned}$$

*Remark* Equations (3) and (5) are consistent if the porous medium consists of a bundle of parallel tubes of radius $R_{eq}$ and porosity $\varepsilon _g$. Indeed, in this case, the permeability *K* is expressed as follows:

7 $$\begin{aligned} K = \frac{\varepsilon _g}{\tau } \left( \frac{R_{eq}^2}{8} \right) \end{aligned}$$

where the tortuosity $\tau$ is equal to the unit for straight tubes.

## Experimental results

### Contact angles

Table 1 summarises the contact angles measured at the macroscopic level in the LR plane of wood. Examples of images obtained with poplar are included in the table. These images and the angle values show that the direction of the image has a very important effect on the measurement. In the radial direction, the surface roughness due to the opening of lumens by the microtome blade artificially limits the spread of the liquid and increases the contact angle. In the present work, the values measured in the longitudinal direction should, therefore, be considered as relevant values and consistent with published data^69^. The effect of the liquid dominates the effect of the species because the contact angles are much smaller for oil than for water. For this reason, a common value will be chosen for each fluid ($18^{\circ }$ for silicone oil and $50^{\circ }$ for water) in the modeling section.

**Table 1:** Contact angles measured for two species (spruce and poplar) for two liquids (water and silicone oil).

Direction	Spruce	Poplar	
**Water**			
L	44.2 (4.6)	52.1 (5.1)	
R	60.1 (5.4)	89.4 (7.5)	
**Silicone oil**			
L	17.1 (4.1)	20.4 (4.3)	
R	25.0 (6.7)	32.0 (5.1)	

Values and (standard deviation) in degrees (photographs by G. Almeida).

### Microscopic imbibition in poplar

**Figure 3:**
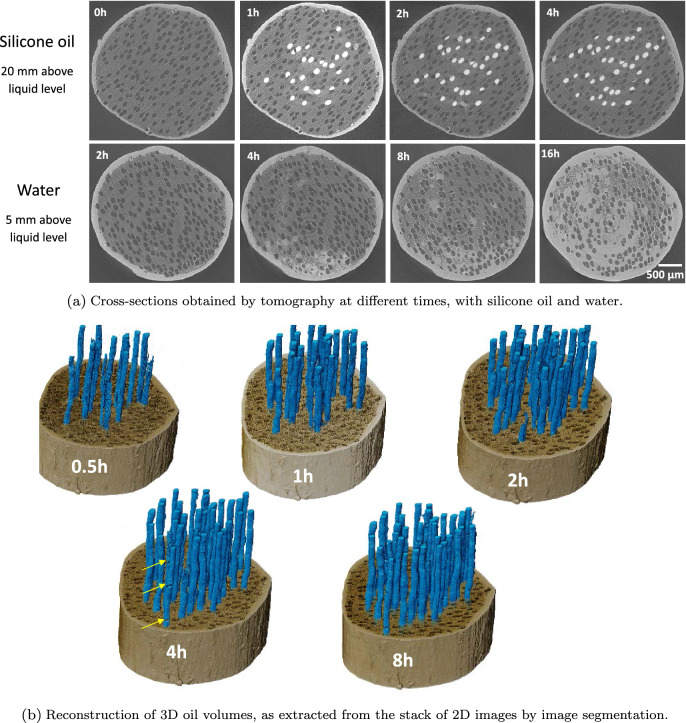
Dynamics of liquid imbibition in poplar: cross sections and 3D reconstruction at different times.

The slices obtained at a constant distance from the exposed face at different times prove that the vessels constitute the main pathways to oil migration in poplar (Fig. 3a, top). Note that not all vessels participate in the transport of silicone oil. As already reported^38^, only a subset of vessels is active, which corresponds to vessel lines open throughout the sample length. Note that absolutely no oil can be observed in fibers, certainly due to the very fine, slit-like pits present in fibers^70^.

**Figure 4:**
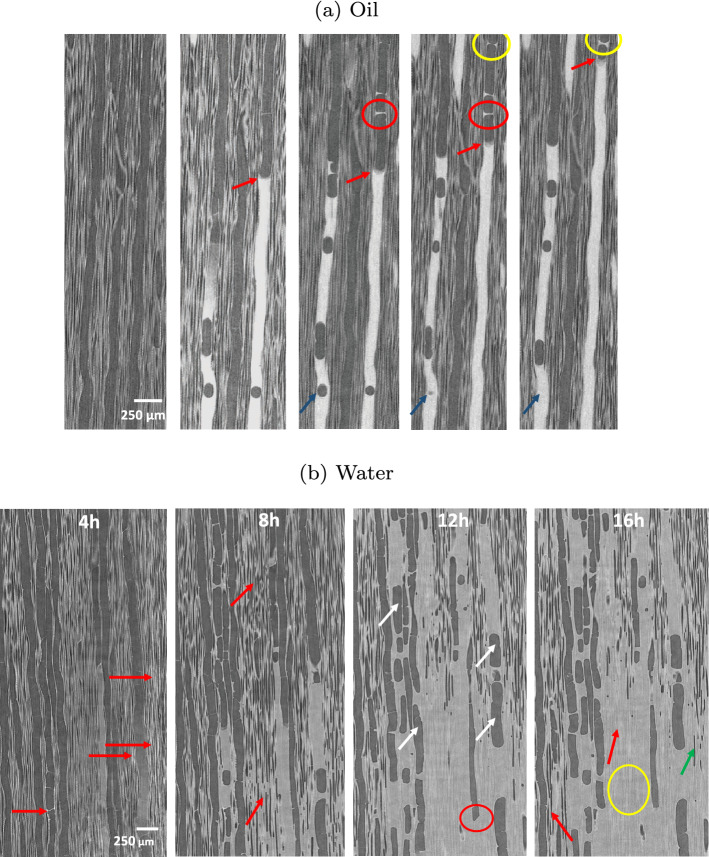
Imbibition in poplar: micro-tomography TL sections at different times.

The contrast of X-ray attenuation between silicone oil and cell wall eases 3D image processing (image segmentation was performed using the software Avizo). Figure 3b confirms that many vessels are not filled with silicone oil whatever the test duration, even though the number of filled vessels increased significantly between 30 min and 2 h. Consistent with our previous comments, these 3D views depict air bubbles that break the silicone oil column (yellow arrows) in the vessels. These air bubbles tend to decrease with time. In conclusion, a very small percentage of vessels are directly connected to the exposed face (ca. 8% at 30 min). This percentage gradually increases to 15% at a longer time due to lateral pathways (certainly in ray cells) allowing unconnected vessels to be filled. The mechanism of silicone oil migration can be further analyzed by TL slices (Fig. 4), which reveal that the silicone oil penetrates in the vessels with a small and homogeneous contact angle, about $30^{\circ }$, consistent with the contact angle measured on macroscopic surfaces. Two complementary phenomena can be observed. At first, the progression of the oil meniscus inside the vessels corresponding to normal capillary behavior in a tube (see red arrows). However, one can also observe trapped air bubbles in vessels, which prove that oil can invade a vessel by a different pathway, such as ray cells. This pathway seems to allow a surface spreading inside the vessels due to the very low contact angle that eventually forms an oil bridge (see red circle), at the position of the circular scar remaining at the junction between two successive vessel cells. Such bridges later develop to the meniscus (see yellow circle).

**Figure 5:**
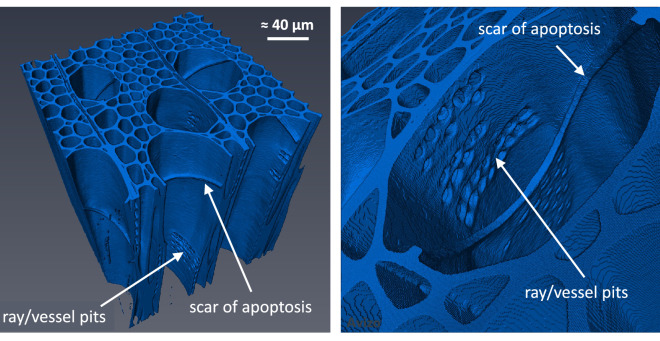
Specific features of poplar anatomy, revealed by a high resolution scan (LaB6 filament of the X-ray nano-source, ca. 8 h scan, 0.3 $\upmu \hbox {m}$ of voxel size).

In contrast to silicone oil, a completely different mechanism is exhibited for water (Fig. 3a, bottom). Indeed, water primarily invaded fibers. This phenomenon may be due to the stronger polarization and surface tension of water than silicone oil, which allows water molecules to react with polarized groups and diffuse through the cell walls of wood and through fiber pits. More surprisingly, most vessels remain empty: the few filled vessels seem to be filled laterally from fibers/ray cells as all filled vessels are close to filled fiber zones. In contrast to silicone oil that penetrated vessels immediately, water migrates in fibers to a quite high level from the short observation times (red arrows at 4 h in Fig. 4b). Even though vessels constitute the vascular system of hardwoods, they are not used for capillary invasion of water in poplar. Over time, water continues to invade fibers (red arrows at 8 h in Fig. 4b) and entire saturated areas develop (yellow circle at 16 h in Fig. 4b). Note that some fibers remain empty at the periphery or even inside these saturated zones (green arrows at 16 h in Fig. 4b). The sequence of images shows that water penetrates fibers first and then some vessels. We can conclude that the contact angle of water (Table 1) is too large for the meniscus to cross the scar between successive vessel cells (Fig. 5). This explanation is confirmed by the relatively high contact angle in vessels, consistent with the angle measured on macroscopic samples, depicted by the images once they contain a water index. Note that, when a meniscus is blocked by a scar, the apparent contact angle inside the vessel can be close to $90^{\circ }$ as reported in^66^. However, this should not be taken as an indication of the actual contact angle.

### Microscopic imbibition in spruce

**Figure 6:**
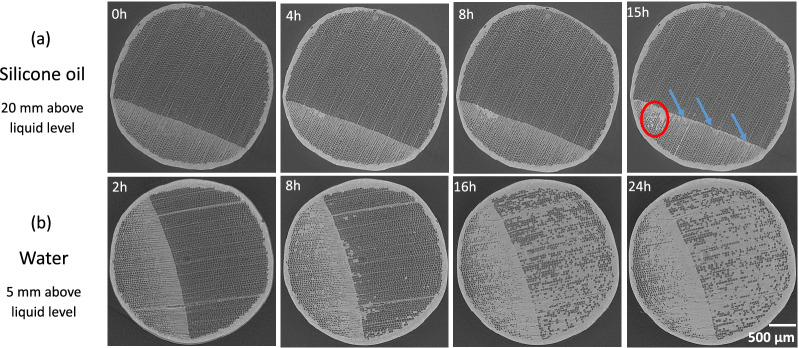
Cross-sections of spruce obtained by tomography at different times during imbibition experiments: (top) with silicone oil and (bottom) with water.

Spruce is composed of about 90% tracheids, which form the main longitudinal liquid pathways in the porous structure. Silicone oil preferably penetrates latewood tracheids with the smallest diameters (Fig. 6a) and then some larger tracheids in the transition wood (red circles at 12 h and 15 h). This observation is classically explained by the low probability of bordered pits aspiration in latewood due to their smaller diameter and larger thickness than earlywood bordered pits^47,71–73^. Like the behavior of oil observed in poplar, it seems that oil cannot travel through narrow or closed pits in spruce, probably as an effect of a non-polar liquid. In the case of water, after the initial longitudinal penetration in latewood, water is then able to gradually spreads to transition wood and earlywood (Fig. 6b). Uniseriate ray cells play a major role in the transverse extension of filled tracheids.

**Figure 7:**
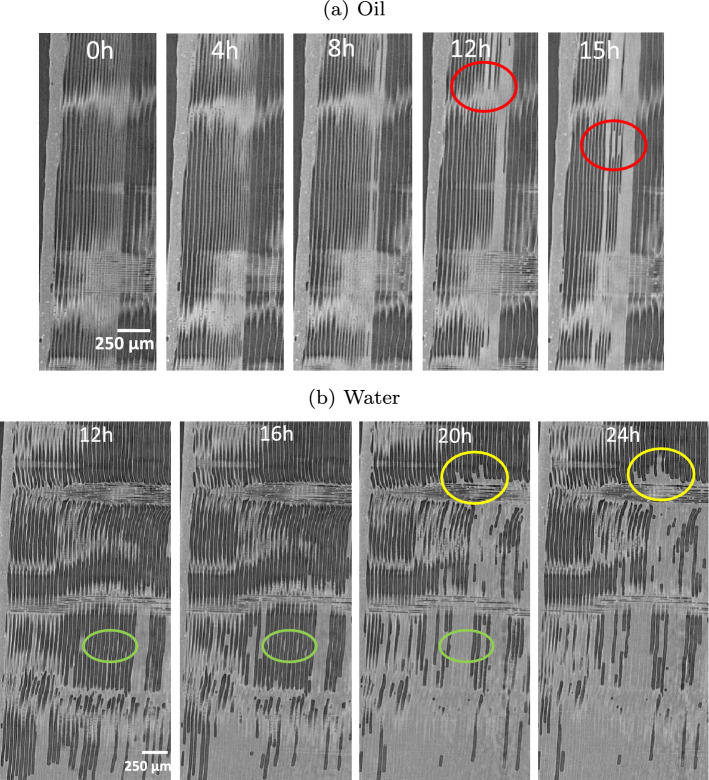
Imbition in spruce: micro-tomography TL sections at different times.

Figure 7a presents the silicone oil imbibition in spruce with different time in TL planes. The liquid progressively invades the latewood. Then, it spreads to some larger-diameter tracheids in latewood or in transition wood nearby latewood over time (see red circles). This lateral extension is possible due to the open bordered pits between them. The possible role of ray cells is not clearly shown in these images. The dynamics of water penetration in spruce are exhibited in TL planes in Fig. 7b. The latewood is on the left-hand side of the figure. This image sequence clearly shows that liquid indexes appear in some tracheids, showing that the migration is not only upwards. These images demonstrate the important role of ray cells in the extension of saturated zones (yellow circle). Water is also likely to migrate from one tracheid to its neighbors through open bordered pits. We also observed that, before the tracheids are filled with water, water droplets of different sizes are formed along their walls. Then those drops develop into large clusters of water and fill the void inside the tracheids (green circles). The air bubbles trapped inside the tracheids by this mechanism decrease or disappear afterward.

### Macroscopic kinetics

**Figure 8:**
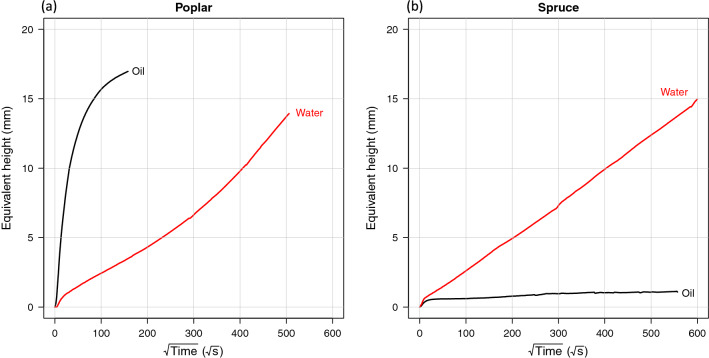
Macroscopic imbibition kinetics obtained for the two species and the two fluids.

The macroscopic dynamics of water and silicone oil imbibition for poplar and spruce in the longitudinal direction are presented as the equivalent height (absorbed liquid volume divided by the sample area) against time (Fig. 8). These macroscopic results depict very different behaviors between species and between liquids. In poplar, oil penetrates rapidly and depicts an asymptotic behavior at around $120^2$ s (ca. 5 h). Imbibition of water is much slower and follows first a linear behavior versus the square root of time. An increasing trend is observed at longer times, from $300^2$ s (ca. 25 h). The trends observed for spruce are completely different: water is faster and follows a quasi-perfect linear behavior throughout the test. The imbibition rate for water is similar to that of poplar. In contrast, the imbibition of oil is much lower than that of water and remains very limited. In the water test, sample swelling due to the increase in bound water was measured in radial (R) and tangential (T) directions every 5 mm along the height (longitudinal direction, L) during the imbibition test. No swelling was observed in the case of oil.

## 1D and 2D modeling

The 1-D Washburn approach described in “Modeling” section was applied to simulate liquid imbibition in poplar and spruce. Four simulations are proposed, with assumptions inferred from the 3D anatomical observations:

- *Oil in vessels* for oil imbibition in poplar; only the porosity of vessels is considered,
- *Oil in latewood* for oil imbibition in spruce; it is assumed that the liquid can only invade the latewood,
- *Water in latewood* to see the time required for water to reach the top of the sample,
- *Water in fibers* for water imbibition in poplar and spruce. The 1D approach assumes the longitudinal permeability to be low due to bordered pits aspiration in spruce and to the small pits opening in poplar. Therefore, a generic “fibers” tissue is proposed in Table 2,

In all simulations, the imbibition was stopped once the height reaches the sample length ($\ell = 100$ mm). The differential Eq. (4) was solved numerically using a second-order Newton explicit method. This computational solution was validated for the case of a sample of sufficient height ($\ell > h_{max}$) using the analytical solution (Eq. 5). This reference solution also allowed the optimal time step to be chosen (low enough to get an accurate solution). Tables 2 and 3 summarize the values used in the simulation. Reasonable orders of magnitude were selected for the different tissues. For example, the permeability of poplar vessels is mid-way between literature data^12,17^ and the theoretical value assuming vessels to be a perfect bundle of straight tubes ($3 \times 10^{-11} m^2$, according to Eq. 7). The latter is smaller, which is classic for hardwood, as the vessels are not tubes of constant diameter, and the probability of connection of vessel lines reduces this theoretical value with the sample length^14,38,74^. The orders of magnitude for spruce latewood and fibers come from theoretical considerations of tissue characteristics together with the probability of bordered pits aspiration^75^.

**Table 2:** Tissue parameters used in Washburn’s model.

Tissues	Permeability (m$^2$)	Porosity (–)	Equivalent radius (m)
Vessels	$5\times 10^{-12}$	0.15	$40\times 10^{-6}$
Latewood	$1\times 10^{-15}$	0.01	$10\times 10^{-6}$
Fibers	$1\times 10^{-14}$	0.2	$10\times 10^{-6}$
Cross-section	$1\times 10^{-18}$	0.5	$20\times 10^{-6}$

**Table 3:** Liquid parameters used in Washburn’s model.

Parameters	Water	Silicone oil
Density (kg m$^{-3}$)	997	1010
Viscosity (Pa s)	0.001	0.02
Surface tension (N s$^{-1}$)	0.073	0.021
Contact angle ($^\circ$)	50	18

**Figure 9:**
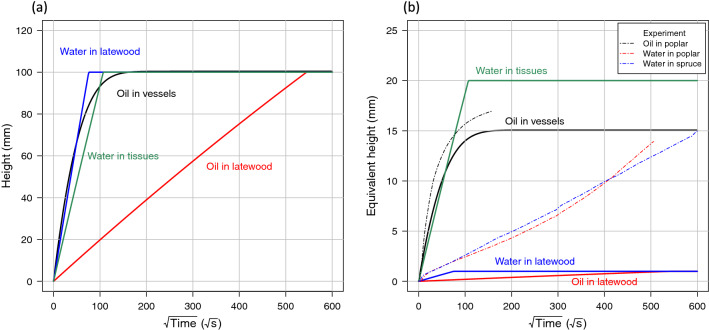
Imbibition modeling using a 1D Washburn formulation. The most important experimental curves are plotted in dashed lines for comparison purposes.

The simulation results (Fig. 9) include two sub-plots: the actual height (left-hand plot) in the anatomical elements, needed to account for the pressure of the liquid column, and the equivalent height (right-hand plot) to be compared with the macroscopic experiments. Both values are related by Eq. (6). The best simulation results are obtained for oil in poplar, for which the 1D model nicely accounts for the time constant and shape of the macroscopic data. The data of Fig. 8 were added to the simulation results for validation purposes. Note that the objective is not to identify the parameters that allow experimental and simulation curves to be superimposed, which is not relevant when considering the biological variability of wood. Instead, we focus on the main trends in this paper. Results are consistent also for oil in spruce: the migration in latewood is very rapid but represents a very small number of pores filled by liquid, hence a very small equivalent height, which is compatible with the experimental observations. Water rises in latewood significantly more rapidly than oil due to the contrasting physical properties of these two fluids: the surface tension (the driving force) is higher in water (0.073 N m$^{-1}$ for water against 0.021 N m$^{-1}$ for oil), and the viscosity (the resistance term) is smaller for water (0.001 Pa s against 0.02 Pa s). However, the amount of water is not significant at the macroscopic level, and the contribution to the equivalent height remains moderate.

Finally, despite realistic values, the 1D simulation of imbibition in fibers, which was expected to represent water imbibition in spruce or poplar, is far too rapid. A longitudinal permeability value of $4\times 10^{-16}\, \mathrm{m}^2$ would have been required to agree with experimental data, which is not realistic in the longitudinal direction. Therefore, the 1D longitudinal Washburn model fails for water. A 2D Washburn model was derived and used to better represent the microscopic observation. Spruce was used for this model as its well-structured anatomical features are simpler to include in a model. This model considers two coupled mechanisms:

- The longitudinal transfer in latewood,
- The lateral spreading of water in earlywood, through rays cells and connected tracheids.

The computational solution requires Eq. (4) to be solved on an evolving 2D network. Kirchoff’s law was applied to each node of this network (conservation of fluxes and equality of pressure). The whole network is then represented in a matrix form. The computational solution of this evolving network was solved by inversion of the updated matrix at each time step. The sample height (100 mm) was divided into 100 discrete elements, which requires less than 1 min of CPU time on a personal computer, with a script written in R (without any compiled function).

**Figure 10:**
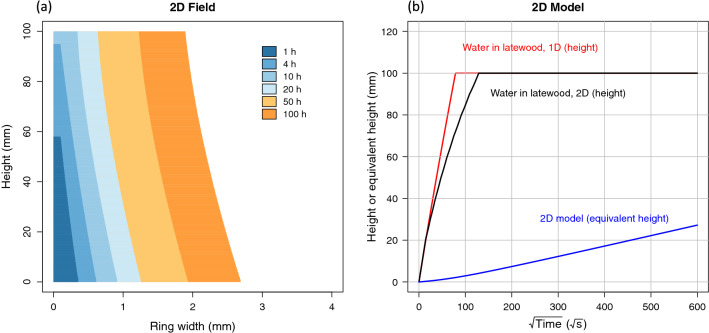
Imbibition modelling of water using a 2D Washburn approach.

The simulation results are depicted in Fig. 10. The 2D fields represent the moisture field over time along the sample height inside one growth ring. As expected, the rise in the latewood is fast: after 1 h, the meniscus level is already at 60 mm, 60% of the sample height. At a given height, lateral spreading starts as soon as the water is present at that level. However, due to the very small permeability, this lateral spreading is very slow despite the small distance (a ring width of 4 mm was considered in this simulation). Once converted in equivalent height, the shape and dynamics of the curve are consistent with the experimental data (Fig. 8b). We also plotted the rise of water in latewood (1D model) and the rise in the same part when latewood must also supply water for lateral extension. Consistently, the lateral flow slows the longitudinal rise in latewood (5 h instead of 2 h to reach 100 mm). The equivalent height assigns all liquid water present in latewood and earlywood, as depicted in the 2D fields, over the sample section. Its time evolution is in complete agreement with the experimental results.

## Conclusion

Imbibition of water and silicone oil in poplar and spruce was investigated at the anatomical level by X-ray tomography observations and macroscopic imbibition kinetics. The anatomical 3-D observations were used to derive relevant assumptions to be embedded in an imbibition model based on the well-known Washburn approach. The macroscopic imbibition kinetics are satisfactorily simulated: a 1D approach is sufficient for oil imbibition while a 2D approach is needed for water. The major conclusions are summarized in Table 4.

**Table 4:** Summary of the main results of this work (graphical illustrations by P. Perré).

Poplar		Spruce	
Silicone oil	Water	Silicone oil	Water
			
Fast rise in vessels. No imbibition in the rest of the structure.	Water meniscus blocked in vessel scars (1). Slow rise/spread through fibers (2) and rays (3). Some vessels supplied in water by rays (4). Vertical rise insured by fibers (5)	Fast rise in latewood (1). No rise or spread in earlywood	Fast rise in latewood (1). Slow rise and spread in earlywood, in connected tracheids (2) and rays (3). Earlywood tracheids participate in vertical rise (4)
Macroscopic measurements are well predicted by a simple Washburn model in vessels	Complex 3D pathways in a heterogeneous pore network. However, the 2D model proposed for spruce gives realistic trends	Washburn’s law applies in latewood	A 2-D Wahsburn model is consistent with the experimental results: initial rise in latewood is then propagation in earlywood

These findings are of crucial importance to propose a suitable continuous macroscopic model able to capture the microscopic phenomena:

- when a 1D Washburn model works, a classical Darcy’s law with a suitable capillary pressure function is advised,
- the need for a 2D model reveals dual-scale phenomena that can still be turned in a macroscopic formulation, provided the dual-scale effects are represented by a relevant memory function able to deal with the failure of local equilibrium^37^.

## Supplementary Information

Supplementary Information.
