# Supplementary material for: A macroscopic Washburn approach of liquid imbibition in wood derived from X-ray tomography observations
Source: Sci Rep. 2022 Feb 2;12:1750. doi: 10.1038/s41598-022-05508-0 (PMC8811000; doi:10.1038/s41598-022-05508-0)
Supplement: Supplementary file 1 — Supplementary Information. [file 41598_2022_5508_MOESM1_ESM.pdf]

# A macroscopic Washburn approach of liquid imbibition in wood derived from X-ray tomography observations

Patrick Perré[a,*], Dang Mao Nguyen[a], Giana Almeida[b]

[a] *Université Paris-Saclay, CentraleSupélec, Laboratoire de Génie des Procédés et Matériaux, SFR Condorcet FR CNRS 3417, Centre Européen de Biotechnologie et de Bioéconomie (CEBB), 3 rue des Rouges Terres 51110 Pomacle, France*

[b] *Université Paris-Saclay, INRAE, AgroParisTech, UMR SayFood, 91300, Massy, France*

## Supplementary material

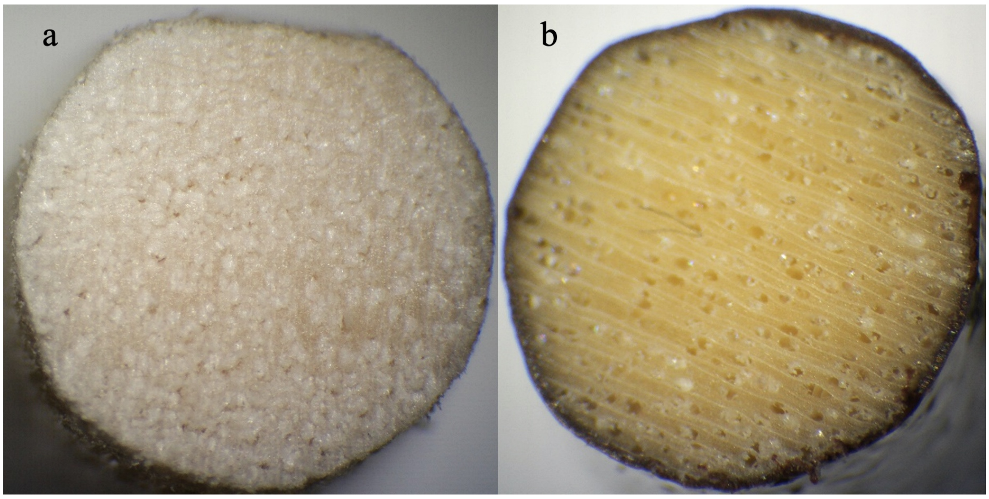


Figure S1: Surface preparation using a sledge microtome prior to imbibition tests : surface of a poplar sample before a) and after b) surface preparation (photographs by M. Nguyen).

[*] Corresponding author

*Email address:* `patrick.perre@centralesupelec.fr` (Patrick Perré )
